# Supplementary material for: Structure and inhibition of Cryptococcus neoformans sterylglucosidase to develop antifungal agents
Source: Nat Commun. 2021 Oct 7;12:5885. doi: 10.1038/s41467-021-26163-5 (PMC8497620; doi:10.1038/s41467-021-26163-5)
Supplement: Supplementary file 1 — Supplementary Information [file 41467_2021_26163_MOESM1_ESM.pdf]

Supplementary Information

Structure and inhibition of *Cryptococcus neoformans* sterylglucosidase to develop antifungals agents

Nivea Pereira de Sa[1], Adam Taouil[2], Jinwoo Kim[2,3], Timothy Clement[2,3], Reece M. Hoffmann[4], John E. Burke[4,5], Robert C. Rizzo[3,6,7], Iwao Ojima[2,3], Maurizio Del Poeta*[1,3,8,9], Michael V. Airola*[3,10].

1. Supplementary Methods

Synthesis of 4-(hydroxymethyl)-1-[2-(3-methoxyphenyl)thiazol-5-yl)methyl]piperidin-4-ol (3: Hit 1).

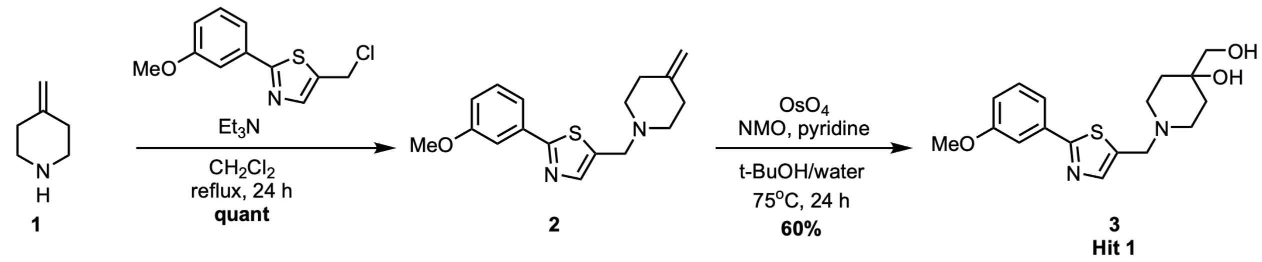

To a solution of 5-(chloromethyl)-2-(3-methoxyphenyl)-1,3-thiazole (780 mg, 3.27 mmol) in dry dichloromethane (33 mL) were added 4-methylidenepiperidine (**1**, 490 mg, 5.03 mmol) and triethylamine (1.1 mL, 8.22 mmol). The reaction mixture was stirred under reflux for 24 hours and then, cooled to room temperature. The reaction was quenched with saturated NH_4Cl aqueous solution (40 mL). The reaction mixture was extracted with dichloromethane (25 mL x 3) and the combined organic layer was washed with water and brine, and then dried over anhydrous $MgSO_4$. The solution was filtered and concentrated under reduced pressure to give 2-(3-methoxyphenyl)-5-(4-methylenepiperidin-1-ylmethyl)thiazole (**2**) (720 mg, quantitative crude yield) as a pale brown oil, which was used for next step without further purification: ^1H NMR (500 MHz, $CDCl_3$) δ 7.60 (s, 1H), 7.49 (m, 1H), 7.47 (d, J = 7.7 Hz, 1H), 7.31 (t, J = 8.0 Hz, 1H), 6.94 (dd, J = 2.2, 8.0 Hz, 1H), 4.65 (d, J = 9.2 Hz, 2H), 3.86 (s, 3H), 3.75 (s, 2H), 2.51 (t, J =5.6 Hz, 4H), 2.25 (t, J = 5.6 Hz, 4H); FIA-MS (ESI) *m/z*: calcd for $C_{17}H_{20}N_2OS$, 300.4, found, $(M+H)^+$ 301.4.

To a solution of **2** (600 mg, 2.00 mmol) in *tert*-butanol (17 mL) were added 4-methylmorpholine *N*-oxide (1.62 g, 13.8 mmol), pyridine (0.85 mL, 10.6 mmol), water (1 mL) and OsO_4 (2.5% in *t*-BuOH, 81.4 μL, 0.008 mmol). The reaction mixture was stirred at 75 ºC for 8 h and then the reaction was quenched with saturated Na_2SO_3 aqueous solution. The reaction mixture was extracted with ethyl acetate and washed with water and brine. Organic layer was dried over

anhydrous $MgSO_4$, filtered and concentrated under reduced pressure to give a crude product. The crude product was purified by flash column chromatography (15% MeOH/CH_2Cl_2) on silica gel to give 4-(hydroxymethyl)-1-((2-(3-methoxyphenyl)thiazol-5-yl)methyl)piperidin-4-ol (**3**: **Hit 1**) (405 mg, 60% yield) as a beige solid: m.p. 113-114 °C; ^{1}H NMR (500 MHz, $CDCl_3$) δ 7.63 (s, 1H), 7.50-7.49 (m, 1H), 7.47 (d, *J* = 7.7 Hz, 1H), 7.32 (t, *J* = 8.0 Hz, 1H), 6.95 (dd, *J* = 1.9, 8.2 Hz, 1H), 3.87 (s, 3H), 3.78 (s, 2H), 3.47 (s, 2H), 2.72 (m, 2H), 2.46 (m, 2H), 1.97 (brs, 2H), 1.65 (m, 4H); ^{13}C NMR (125 MHz, $CDCl_3$) δ 168.4, 160.0, 141.9, 136.6, 135.0, 130.0, 119.0, 116.4, 110.8, 70.3, 69.6, 55.4, 54.5, 48.8, 33.7; HRMS (ESI-TOF) *m/z* calcd for $C_{17}H_{22}N_2O_3S$ $[M+H]^+$ 335.1430, found 335.1424 (Δ = -1.79 ppm). Purity analysis by HPLC: 100% at 210 and 254 nm.

Synthesis of *N*-[(3*R*,5*S*)-5-(hydroxymethyl)-1-methylpyrrolidin-3-yl)-2-(3-oxo-3,4-dihydro-2*H*-benzo[b][1,4]oxazin-6-yl)acetamide (10: Hit 9)

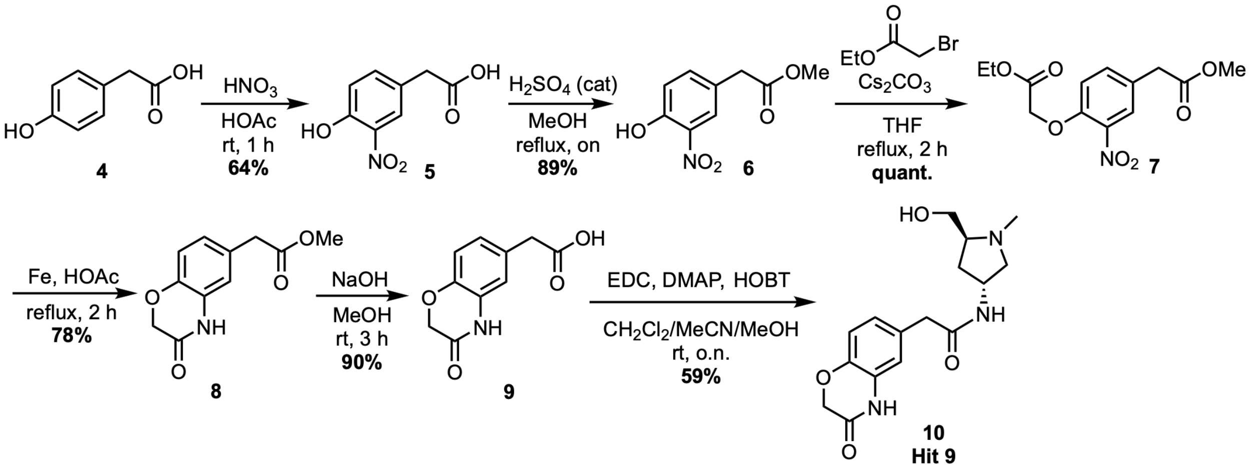

To an ice-cooled solution of 4-hydroxyphenylacetic acid (**4**) (10.0 g, 6.6 mmol) in acetic acid (50 mL) was added dropwise 70% nitric acid (4.4 mL) in acetic acid (5 mL) with stirring, wherein the temperature was kept a below 10 °C. The resulting yellow solution was stirred for 1 h at room temperature, during which brown started precipitating, and then the reaction mixture was cooled in an ice-bath. The precipitates were collected by filtration and washed with water to give yellow soid, which was recrystallized from ethyl acetate/hexanes to give 2-(4-hydroxy-3-nitrophenyl)acetic acid (**5**) (8.03 g, 64% yield) as a yellow solid: ^{1}H NMR (500 MHz, $CDCl_3$) δ 12.27 (s, 1H), 8.03 (d, *J* = 2.0 Hz, 1H), 7.51 (dd, *J* = 2.0, 8.6 Hz, 1H), 7.15 (d, *J* = 8.6 Hz, 1H), 3.66 (s, 2H).

To a solution of **5** (5.21 g, 26.4 mmol) in methanol was added conc. H_2SO_4 (0.7 mL), and the reaction mixture was heated under reflux with stirring overnight. The reaction mixture was then cooled to room temperature and the solvent removed under reduced pressure. The residue was neutralized with saturated $NaHCO_3$ and the aqueous reaction mixture was kept in a

refrigerator overnight. The resulting precipitate was collected by filtration, washed with deionized water, and dried *in vacuo* to give methyl 2-(4-hydroxy-3-nitrophenyl)acetate (**6**) (4.95 g, 89% yield) as a yellow solid: ^{1}H NMR (700 MHz, DMSO) δ 10.90 (s, 1H), 7.83 (d, *J* = 2.1 Hz, 1H), 7.45 (dd, *J* = 8.5, 2.2 Hz, 1H), 7.09 (d, *J* = 8.5 Hz, 1H), 3.71 (s, 2H), 3.63 (s, 3H). ^{13}C NMR (176 MHz, DMSO) δ 171.94, 151.51, 137.03, 136.76, 126.19, 126.08, 119.50, 52.27, 38.72. These data are consistent with literature values.[1]

To a suspension of **6** (4.95 g, 23.4 mmol) and cesium carbonate (21.5 g, 46.8 mmol) in dry THF (20 mL) was added dropwise a solution of ethyl 2-bromoacetate (3.1 mL, 28.1 mmol) in dry THF (15 mL) with stirring at room temperature. The resulting solution was heated under reflux for 2 hours. The reaction mixture was cooled to room temperature and the reaction was quenched with deionized water (30 mL). The reaction mixture was extracted with ethyl acetate (3 x 30 mL). The combined organic layers were dried over anhydrous $MgSO_4$, filtered, and concentrated *in vacuo* to give a crude product. The crude product was purified on silica gel with 5-40% ethyl acetate in hexanes as eluent to give ethyl 2-(4-(2-methoxy-2-oxoethyl)-2-nitrophenoxy)acetate (**7**) (6.96 g, quantitative yield) as a yellow solid: ^{1}H NMR (700 MHz, $CDCl_3$) δ 7.83 (d, *J* = 2.1 Hz, 1H), 7.46 (dd, *J* = 8.7, 2.1 Hz, 1H), 6.97 (d, *J* = 8.6 Hz, 1H), 4.78 (s, 2H), 4.30 – 4.25 (m, 2H), 3.73 (s, 3H), 3.64 (s, 2H), 1.30 (t, *J* = 7.1 Hz, 3H). ^{13}C NMR (176 MHz, $CDCl_3$) δ 171.01, 167.74, 150.48, 134.95, 127.87, 126.69, 115.46, 66.68, 61.77, 52.35, 39.61, 14.10. These data are consistent with literature values.[2]

To a solution of **7** (5.02 g, 16.9 mmol) in acetic acid (30 mL) was added iron powder (2.81 g, 50.7 mmol) in several portions. The reaction mixture was heated under reflux overnight overnight, cooled to room temperature, and the solid was filtered over celite. The filtrate was diluted with deionized water (20 mL) and extracted with ethyl acetate (3 x 30 mL). The combined organic layers were washed with brine (30 mL), dried over anhydrous $MgSO_4$, and the solvent was removed *in vacuo* to give methyl 2-(3-oxo-3,4-dihydro-2*H*-benzo[b][1,4]oxazin-6-yl)acetate (**8**) (2.92 g, 78% yield) as a crude product, which was used in the next step without further purification: ^{1}H NMR (700 MHz, DMSO) δ 10.70 (s, 1H), 6.90 – 6.87 (m, 1H), 6.82 – 6.77 (m, 2H), 4.55 (d, *J* = 4.3 Hz, 1H), 3.60 (s, 2H). ^{13}C NMR (176 MHz, DMSO) δ 172.51, 172.10, 165.42, 142.67, 128.88, 127.63, 124.39, 117.04, 116.45, 67.20, 52.16. These data are consistent with literature values.[2]

To a solution of **8** (2.50 g, 11.3 mmol) in methanol (25 mL) was added a 1 M solution of sodium hydroxide (30 mL). The resulting solution was stirred for 3 hours at room temperature, and the organic solvent was removed under reduced pressure. The reaction mixture was acidified with 2M HCl to pH ~3. The resulting precipitate was collected by filtration, washed with deionized water and dried in vacuo to give 2-(3-oxo-3,4-dihydro-2*H*-benzo[b][1,4]oxazin-6-yl)acetic acid

(**9**) (2.11 g, 90%) as a gray solid: ^{1}H NMR (700 MHz, DMSO) δ 12.32 (s, 1H), 10.70 (s, 1H), 6.88 (d, *J* = 7.8 Hz, 1H), 6.79 (d, *J* = 8.3 Hz, 2H), 4.54 (s, 2H), 3.47 (s, 2H); ^{13}C NMR (176 MHz, DMSO) δ 173.19, 165.46, 142.51, 129.60, 127.53, 124.41, 117.08, 116.33, 67.22. These data are consistent with literature values.[2]

To a solution of **9** (500 mg, 2.41 mmol), 1-ethyl-3-(3-dimethylaminopropyl)carbodiimide hydrochloride (EDC·HCl) (449 mg, 2.89 mmol), hydroxybenzotriazole (HOBT) (391 mg, 2.89 mmol), and 4-dimethylaminopyridine (DMAP) (30 mg, 0.25 mmol) in acetonitrile (20 mL) was added a solution of (2*S*,4*R*)-(4-amino-1-methylpyrrolidin-2-yl)methanol (375 mg, 2.90 mmol) in acetonitrile (10 mL), and the reaction mixture was allowed to stir overnight at room temperature. The solvent was removed *in vacuo* from the reaction mixture, the residue taken up in water (20 mL), and extracted with ethyl acetate (3 x 20 mL). The combined organic layers were washed with brine (20 mL), dried over anhydrous $MgSO_4$, and concentrated *in vacuo* to give a crude product as an oil. The crude product was first purified on C_{18} silica gel, using a gradient of acetonitrile (0-30%) in water. To remove residual HOBT, the product was further purified on a normal phase silica gel column using MeOH:CH_2Cl_2:NEt_3 (20:79:1) as eluent to give *N*-[(3*R*,5*S*)-5-(hydroxymethyl)-1-methylpyrrolidin-3-yl)-2-(3-oxo-3,4-dihydro-2*H*-benzo[b][1,4]oxazin-6-yl]-acetamide (**10**, **HIT 9**) (450 mg, 59% yield) as a white solid: m.p. 224-225 °C (decomp.); $[\alpha]_D^{20}$ -12.74 (*c* = 0.16 in MeOH); ^{1}H NMR (700 MHz, DMSO-*d*$_6$) δ 10.70 (s, 1H), 8.13 (d, J = 7.2 Hz, 1H), 6.86 (d, J = 8.1 Hz, 1H), 6.82 – 6.73 (m, 2H), 4.52 (s, 2H), 4.44 (t, J = 5.3 Hz, 1H), 4.04 (dd, J = 15.6, 8.5 Hz, 1H), 3.43 – 3.37 (m, 1H), 3.27 (s, 2H), 3.27 – 3.21 (m, 1H), 3.13 (dd, J = 8.5, 7.0 Hz, 1H), 2.38 (s, 1H), 2.26 (s, 3H), 2.00 (t, J = 8.6 Hz, 1H), 1.85 (ddd, J = 13.0, 9.1, 7.2 Hz, 1H), 1.64 (ddd, J = 13.0, 8.5, 6.5 Hz, 1H). ^{13}C NMR (176 MHz, DMSO-*d*$_6$) δ 170.24, 165.50, 142.31, 131.08, 127.44, 123.92, 116.68, 116.26, 67.22, 66.06, 63.78, 63.12, 47.09, 41.92, 41.34, 35.86; HRMS (ESI-TOF) *m/z* calcd for $C_{16}H_{22}N_3O_4^+$ [M+H]$^+$, 320.1605, found, 320.1600, (Δ = 1.56 ppm). Purity analysis by HPLC: 100% at 210 and 254 nm.

Synthesis of [(2*S*,4*R*)-4-(6-isopropyl-1-methyl-1*H*-pyrazolo[3,4-*d*]pyrimidin-4-ylamino)-1-methylpyrrolidin-2-yl]methanol (Hit 15)

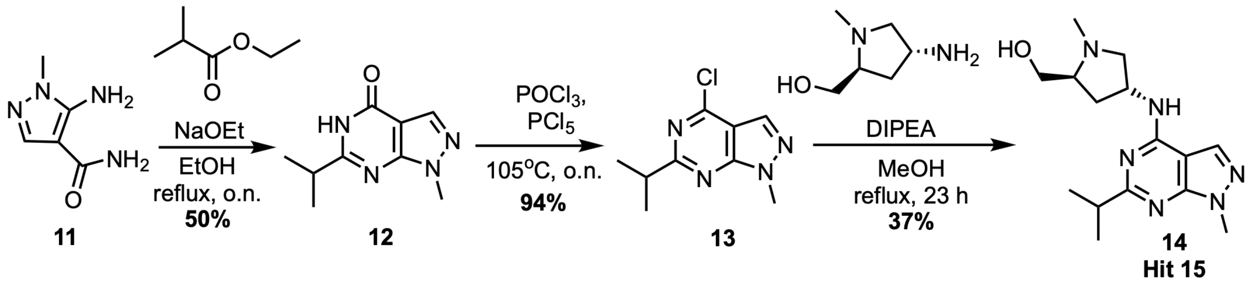

5-Amino-1-methyl-1*H*-pyrazole-4-carboxamide (**11**) was prepared from commercially available ethoxymethylenemalononitrile by the literature method.[3] To a mixture of **11** (1.48 g, 10.52 mmol) and sodium ethoxide in ethanol (15 mL, 40.2 mmol, 21 wt. % in ethanol) was added ethyl isobutyrate (2.7 mL, 20.1 mmol), and the mixture was heated under reflux overnight. The reaction mixture was cooled to room temperature, and concentrated to dryness *in vacuo*. The resulting crude product was dissolved in water (15 mL) and then filtered to remove solid impurities. The filtrate was neutralized with conc. HCl to pH 6-7 to cause precipitation. Then, the precipitate was collected by filtration and recrystallized from MeOH to give 6-isopropyl-1-methyl-1*H*-pyrazolo[3,4-*d*]pyrimidine (**12**) (1.02 g, 50% yield) as a yellow solid: m.p. >230^oC; ^1H NMR (700 MHz, DMSO) δ 11.98 (s, 1H), 7.98 (s, 1H), 3.88 (s, 3H), 2.98 – 2.89 (m, 1H), 1.25 (d, *J* = 6.9 Hz, 6H); ^{13}C NMR (176 MHz, DMSO) δ 165.46, 158.61, 152.78, 134.35, 104.38, 34.29, 33.52, 20.89. These data are consistent with literature values.[3]

To a solution of **12** (710 mg, 3.67 mmol) was in $POCl_3$ (10 mL) was added PCl_5 (760 mg, 3.67 mmol) and the mixture was heated under reflux with stirring overnight. The reaction mixture was cooled to room temperature and concentrated under reduced pressure. The residue was quenched with ice water, extracted with ethyl acetate and washed with sat. $NaHCO_3$ and brine. The combined organic layers were dried over anhydrous $MgSO_4$ and filtered. The filtrate was concentrated in vacuo to give 4-chloro-6-isopropyl-1*H*-pyrazolo[3,4-*d*]pyrimidine (**13**) (730 g, 94% yield) as the crude product, which was used in the next step without further purification: ^1H NMR (300 MHz, $CDCl_3$) δ 8.06 (s, 1H), 4.11 (s, 3H), 3.33-3.17 (m, 1H), 1.39 (d, *J* = 6.9 Hz, 6H).

To a solution of **13** (730 mg, 3.46 mmol) in methanol (35 mL) were added (2*S*,4*R*)-(4-amino-1-methylpyrrolidin-2-yl)methanol (540 mg, 4.13 mmol) and *N,N*-diisopropylethylamine (DIPEA) (0.72 mL, 4.13 mmol) subsequently. The resulting mixture was heated under reflux with stirring for 23 h. The reaction mixture was concentrated under reduced pressure to give a crude product, which was purified by column chromatography on silica gel (AcOEt:hexanes) to give [(2*S*,4*R*)-4-(6-isopropyl-1-methyl-1*H*-pyrazolo[3,4-*d*]pyrimidin-4-ylamino)-1-methylpyrrolidin-2-yl]methanol (**Hit 15**, 390 mg, 37% yield) as a brownish oil: ^1H NMR (500 MHz, $CDCl_3$) δ 7.86 (s, 1H), 5.68 (brs, 1H), 4.65 (brs, 1H), 3.97 (s, 3H), 3.73 (d, J = 11.1 Hz, 1H), 3.66 (m, 1H), 3.50 (d, J = 10.3 Hz, 1H), 3.46 (s, 1H), 3.03-2.97 (m, 1H), 2.74 (brs, 1H), 2.46-2.38 (m, 5H), 1.92-1.86 (m, 1H), 1.29 (d, J = 6.8 Hz, 6H); ^{13}C NMR (125 MHz, $CDCl_3$) δ 172.9, 156.5, 154.4, 130.5, 98.8, 65.3, 63.1, 61.1, 50.0, 49.2, 40.2, 37.5, 34.9, 33.6, 21.7, 21.7. HRMS (ESI) *m/z* calcd for $C_{15}H_{24}N_6O$ $[M+H]^+$ 305.2084, found 305.2081 (Δ = 1.08 ppm). Purity analysis by HPLC: 98.9% at 210 nm and 98.9% at 254 nm.

2. **Supplementary Tables**

Supplementary Table 1. Small molecule screening data.

Category	Parameter	Value/ Description	Additional information
Assay	Plate format	96-well	Solid black polystyrene microplate, round bottom
	Reaction buffer	pH 6	50 mM citric acid, 176 mM K_2HPO_4, 0.01% Tween-20 and 10 mM sodium taurocholate (Urban et al., 2008)
	Substrate	10 µL	100 µM stock solution
	Enzyme	17 µL	Containing 20 ng of Sgl1
	Incubation time	30 min	37°C, 5% CO_2
Library	Size	50,000	ChemBridge DIVERSet-CL library
	Cocktail preparation	5000	10 compounds/well at 10 µM each (10% DMSO in DPBS)
Screen	Concentrati on tested	1 µM	3 µL/well of a 10 µM stock
	Controls	3 µL	Positive control (DPBS) and DMSO control (10% DMSO in DPBS)
	Readout	570 (±10) / 610 (±10) nm	VersaMax™ Microplate Reader
Post-HTS analysis	Cocktails hits criteria	>50% inhibition	Cocktails hits were categorized by percentage of inhibition
	Individuals hits criteria	>50% inhibition	5 µM
	Hits confirmation	0.25-16 µM	Dose-response evaluation
	Hit rate	0.03%	15 inhibitors identified from the library
	Additional tests	Toxicity against mammalian cell line A549	Only hits with IC50 <2 were evaluated. Hits with selectivity index (SI) higher than 500 were selected
	Additional comments	Top 3 hits production	Synthesized by Dr. Iwao Ojima laboratory at Stony Brook University

Supplementary Table 2. Fifteen single compound hits identified by HTS.

Hit N°	Chembridge ID	Structure	Mol Formula	Mol Weight	cLogP	IC50 (μM)	LD50 (μM)	SI
1	59928901	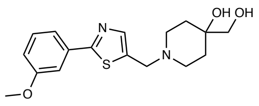	$C_{17} H_{22} N_2 O_3 S$	334.4	1.49	1	655	655
2	52677391	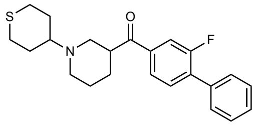	$C_{23} H_{26} F N O S$	383.5	4.963	4	NT	NA
3	26113857	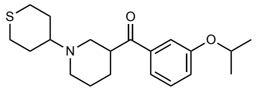	$C_{20} H_{29} N O_2 S$	347.5	3.915	4	NT	NA
4	13077737	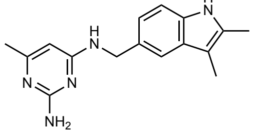	$C_{16} H_{19} N_5$	281.4	3.49	4	40.4	10.1
5	24390922	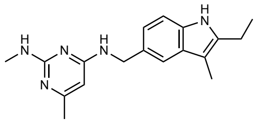	$C_{18} H_{23} N_5$	309.4	2.96	1	50	50
6	70625939	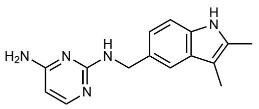	$C_{15} H_{17} N_5$	267.3	1.56	2	181.3	90.65
7	98977858	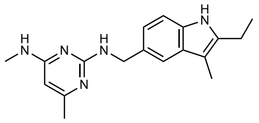	$C_{18} H_{23} N_5$	309.4	3.21	2	87	43.5

8	68640853	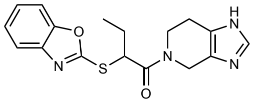	$C_{17} H_{18} N_4 O_2 S$	342.4	2.95	4	NT	NA
9	23645796	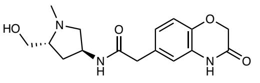	$C_{16} H_{21} N_3 O_4$	319.4	-0.583	0.5	585.3	1170.6
10	47515663	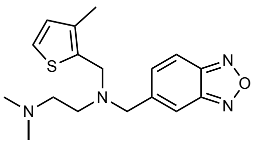	$C_{17} H_{22} N_4 O S$	330.4	3.858	0.5	301.8	603.6
11	41399995	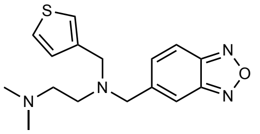	$C_{16} H_{20} N_4 O S$	316.4	3.409	4	NT	NA
12	51897881	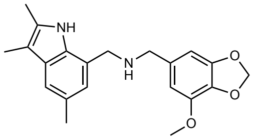	$C_{21} H_{24} N_2 O_3$	352.4	3.662	2	NT*	NA
13	46415405	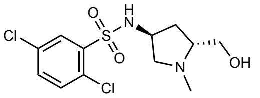	$C_{12} H_{16} Cl_2 N_2 O_3 S$	339.2	2.278	2	217.3	108.65
14	61955612	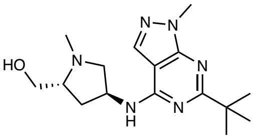	$C_{16} H_{26} N_6 O$	318.4	1.25	1	357	357
15	86711567	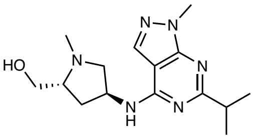	$C_{15} H_{24} N_6 O$	304.4	0.91	1	>512	512

3. Supplementary Data

Supplementary Figure 1. Kinetic analysis of substrates ergosterol 3β-D-glucoside (erg-glc) and C6-NBD-glucosylceramide (C6-NBD-GlcCer). **a.** System linearity of ergosterol detection at 282 nm by UV absorption after HPLC separation. **b.** Enzyme linearity of reactions using 1 mol% erg-glc in Triton-X-100 mixed micelles with 12 min reaction time at 37°C. **c.** Time linearity of reactions using 1 mol % erg-glc and 52.8 fmol of Sgl1 at 37°C. **d.** System linearity of C6-NBD-Ceramide (C6-NBD-Cer) detection by fluorescence (Ex./Em. 470/530 nm) after HPLC separation. **e.** Enzyme linearity of reactions using 2.5 mol% of C6-NBD-GlcCer in Triton-X-100 mixed micelles with 35 min reaction time at 37°C. **f.** Reaction time of Sgl1 and C6-NBD-GlcCer is linear until 60 minutes using 2.5 mol % of substrate and 1.58 pmol of Sgl1 at 37°C.

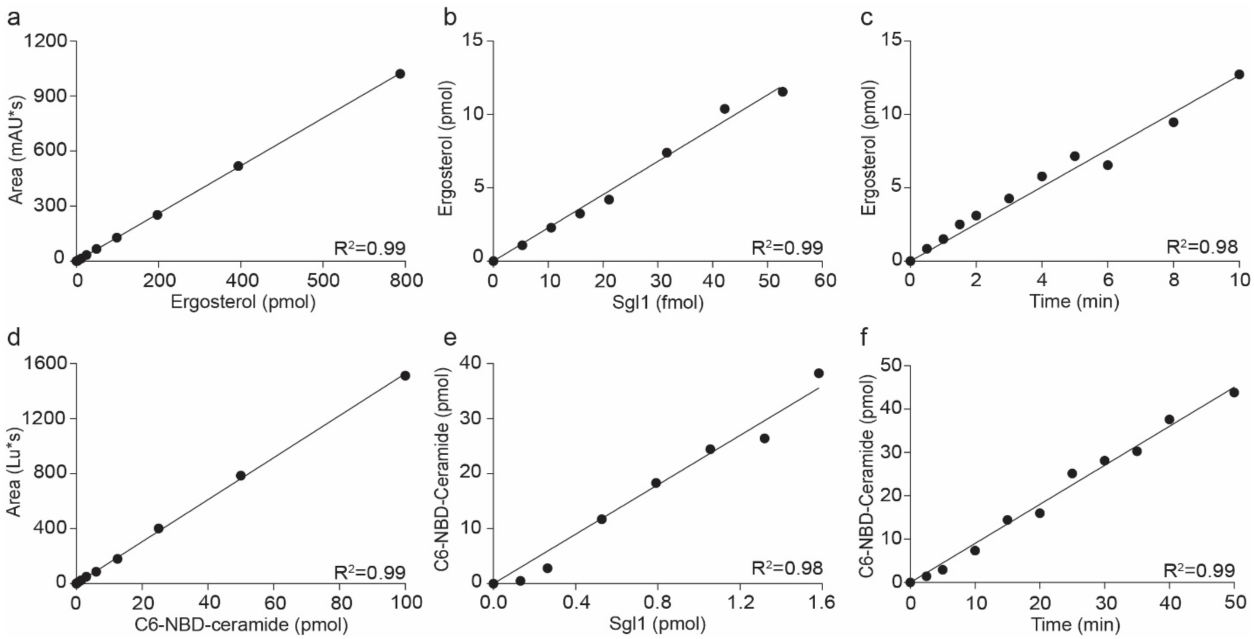

Supplementary Figure 2. a. % deuterium incorporation after a 3 sec deuterium exposure at 1°C. Each point represents a single peptide (from 264 total peptides), with them being graphed on the x-axis according to their central residue. %D was corrected for back exchange as described in methods, and %D represents absolute %D incorporation. This is represented by a heat map above, which is color coded according to the legend. The experiment identified three regions with deuterium incorporation rates higher than 90%, indicating disordered segments near residues 609-634, 723-777, and at the C-terminus. Data are presented as mean values +/- SD of n=3 samples from a single experiment. **b.** Domain architecture of wild type Sgl1 and three constructs with deletions on the disordered regions. All three Sgl1 retained full enzymatic activity against ergosterol 3β-D-glucoside. Data represent the mean values +/- SD of n=3 independent experiments. Statistical analysis by one-way ANOVA, Dunnett's Multiple Comparison Test.

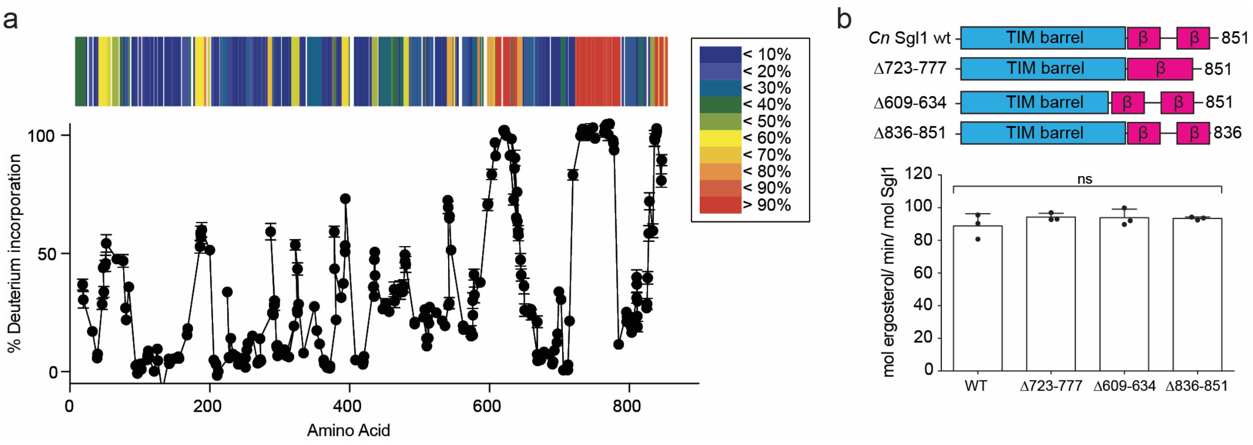

Supplementary Figure 3. Structure-based sequence alignment of Sgl1 homologs. Identical residues shaded red, homologous residues shaded yellow. Secondary structure elements for the *Cn* Sgl1 structure are indicated above, “TT” = turn; Mutated residues predicted to bind glucose (*) or within the hydrophobic pocket (black diamond) are indicated beneath.

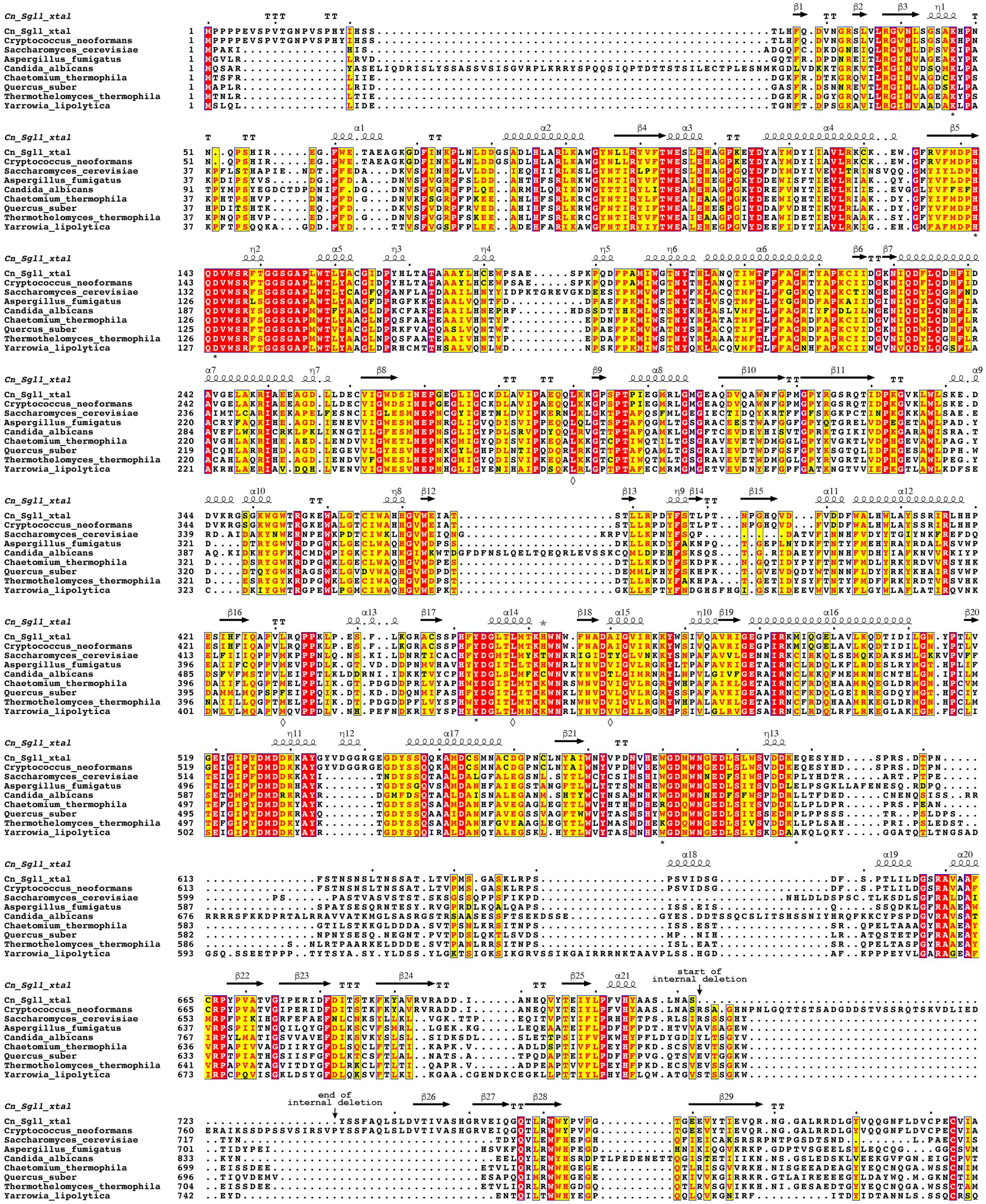

Supplementary Figure 4. Crystal structure of Sgl1 with a tris molecule bound in the active site (left panel). The tris molecule interacts with residues Glu520, Glu270, Lys47, Tyr453, and Glu587 of Sgl1. Crystal structure of cerezyme also determined with a tris molecule bound in the active site (right panel) in a different conformation as observed in Sgl1. The tris molecule interacts with both catalytic glutamate residues, and with Asp127, Asn396, Tyr244, which are residues that differ from the Sgl1 active site.

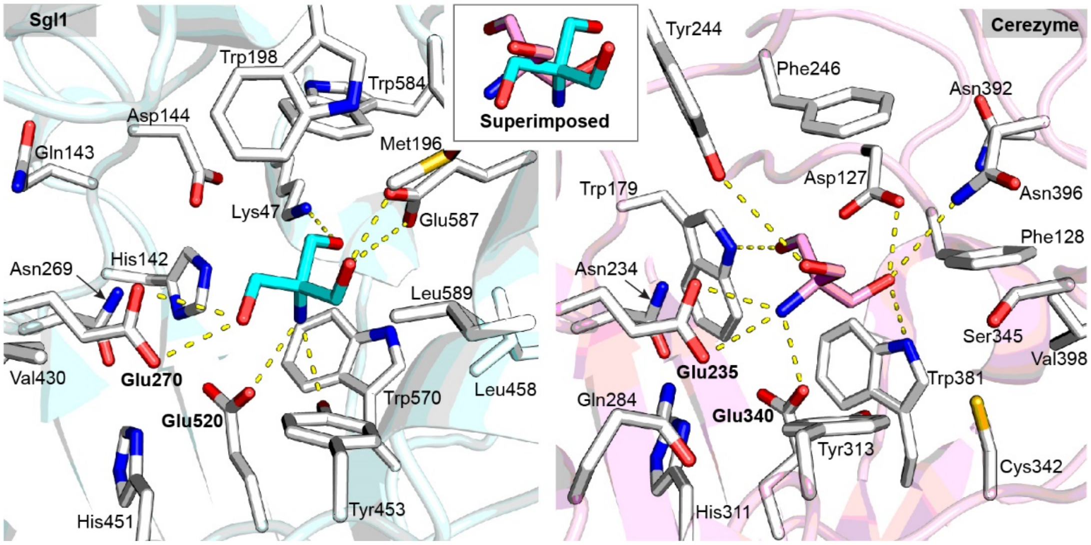

Supplementary Figure 5. Glucosylceramide docking comparison at the Sgl1 active site. C6-NBD-glucosylceramide (C6-NBD-GlcCer, yellow) sugar moiety is positioned the same way as erg-glc displayed in Figure 3a. C6-NBD-GlcCer fit well within the Y-shaped pocket with the C6-acyl-NBD-group located in the buried hydrophobic cavity where erg-glc binds and the sphingosine chain branching into the solvent exposed section of the Y-shaped cavity. Fungal glucosylceramide (green) has a hydroxyl group that would generate steric clashes with residue Glu270, which obliges the lipid to contort, thus moving the glucose to a non-canonical position.

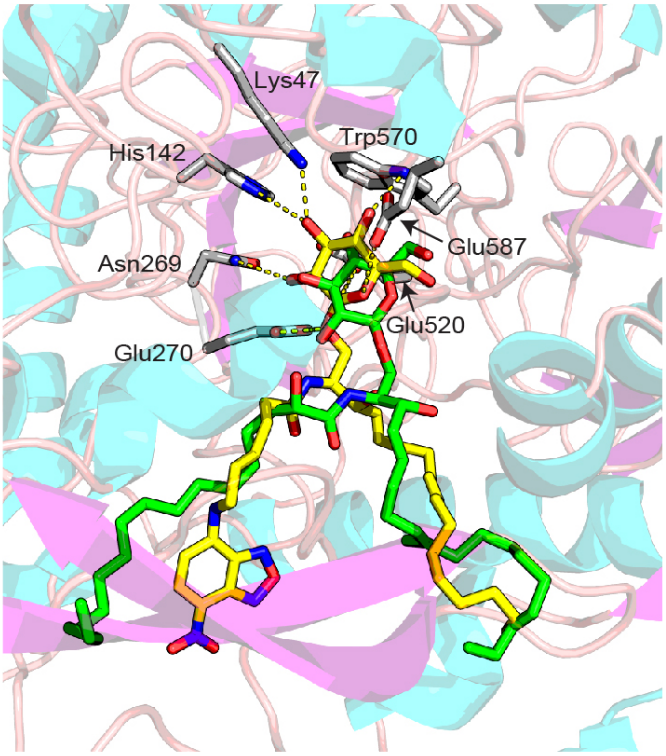

Supplementary Figure 6. High-throughput screening assay parameters. **a.** Resorufin 3β-D-glucopyranoside (res-glp) hydrolysis by Sgl1 releases the fluorescent product resorufin and glucose. **b.** Detection linearity of the system. **c.** Temperature evaluation of Sgl1 activity (n=6). **d.** Reaction dependence on pH (n=4). **e.** DMSO tolerance (n=6). **f.** Enzyme concentration linearity (n=4). The reaction is linear up to 0.4 pmol of Sgl1 in presence of the substrate res-glp (100 μM). Data presented as mean values +/- SD of 2-3 independent experiments performed in duplicate (c-f). **g.**Time linearity until 50 min (n=4). **h.** Eight substrate concentrations tested with 0.2 pmol of Sgl1 until 20 min. **i.** Kinetic curve of Sgl1 (n=4). Error bars represent standard deviation of four independent experiments performed in technical duplicates. **j.** Kinetic parameters of Sgl1 reaction with res-glp.

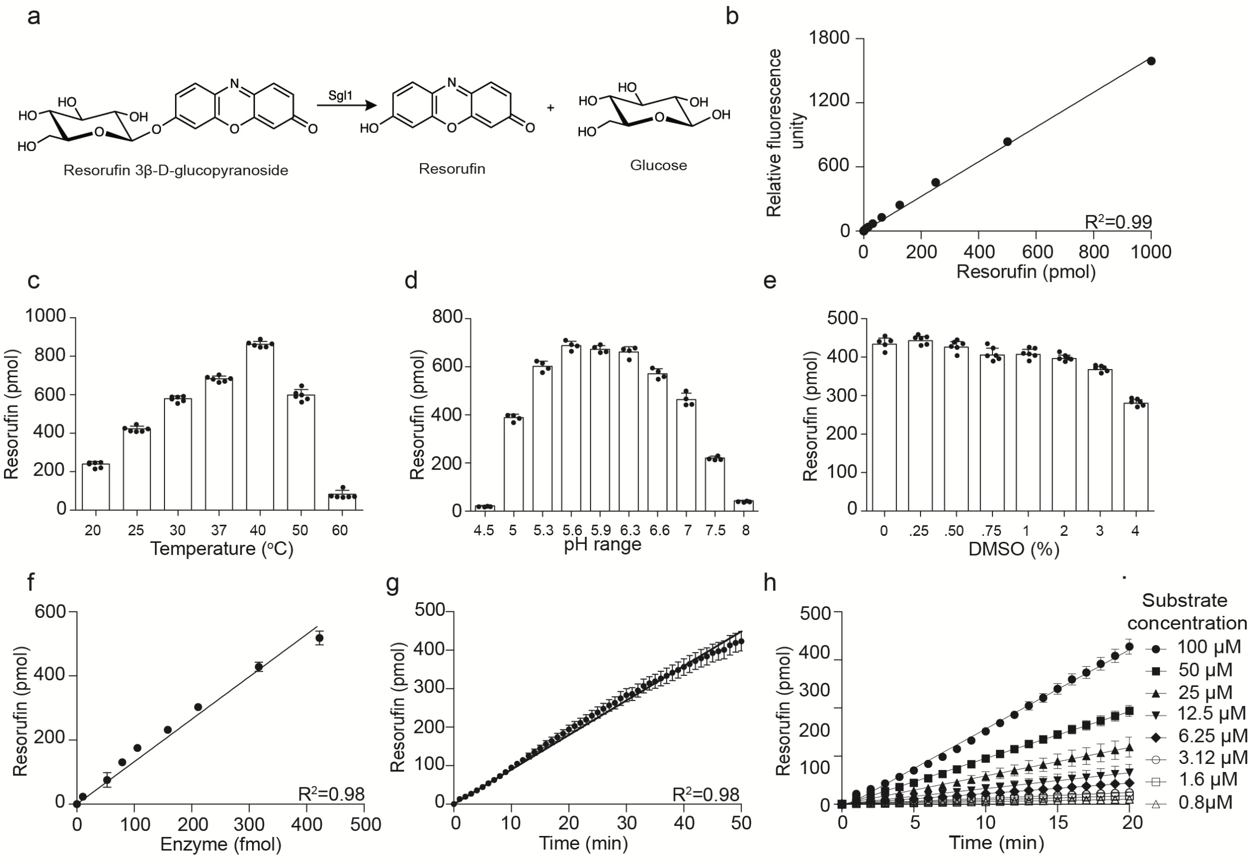

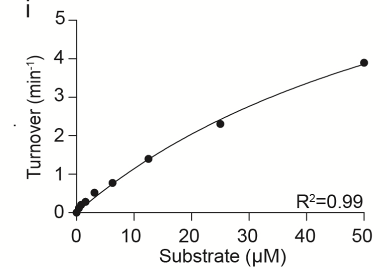

j

Substrate	K_M (μM)	k_{cat} (min^{-1})	k_{cat}/K_M (min^{-1} M^{-1})
Resorufin 3β-D-glucopyranoside	146.8 ± 26.35	3.89 ± 0.03	2.65 ± 0.11 x 10^{-2}

Supplementary Figure 7. Analysis of the purity of re-synthesized and verified Hits 1, 9, and 15 by LC-UV-MS

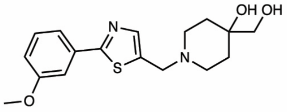

Hit 1
Chemical formula: $C_{17}H_{22}N_2O_3S$
Exact mass: 334.13511
Log P: 1.49
Log S: -2.053
pka: 7.625, 13.883, 17.965

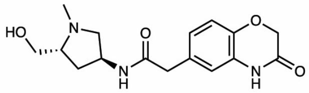

Hit 9
Chemical formula: $C_{16}H_{21}N_3O_4$
Exact mass: 319.15321
Log P: -1.22
Log S: -1.343
pka: 7.937, 14.606

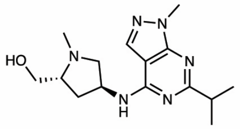

Hit 15
Chemical formula: $C_{15}H_{24}N_6O$
Exact mass: 304.20116
Log P: 1.72
Log S: -2.287
pka: 8.113, 14.644

Hit N°	Rt (min)	210 nm area%	254 nm area %	MS-TIC area %	$[M+H]^+$ meas	$[M+H]^+$ calc	Error (ppm)
1	5.76	100	100	100	335.1430	335.1424	-1.88
9	4.32	100	100	100	320.1600	320.1605	1.66
	5.20	0.4	0.3	1.4	291.1929		
15	5.50	98.3	98.9	96.3	305.2081	305.2084	1.08
	5.78	1.3	0.8	2.3	303.1929		

Supplementary figure 8. Cerezyme is not inhibited by its 1, 9 and 15. 10 µg of Cerezyme was added to 10 µg of mammalian C18-glucosylceramide in the presence of 100 µM of compound hits. The reactions were performed in 50 mM sodium acetate buffer pH5.5 and incubated at 37°C for 3 hours. The amount of C18-ceramide formed was quantified by LC-MS. There was no significant difference between the control (no drug) and samples with 100 µM of hits 1, 9 and 15 ($p>0.05$). Statistical analysis by one-way ANOVA, Dunnett's Multiple Comparison Test. Data represent the mean +/- SD of n=3 independent experiments.

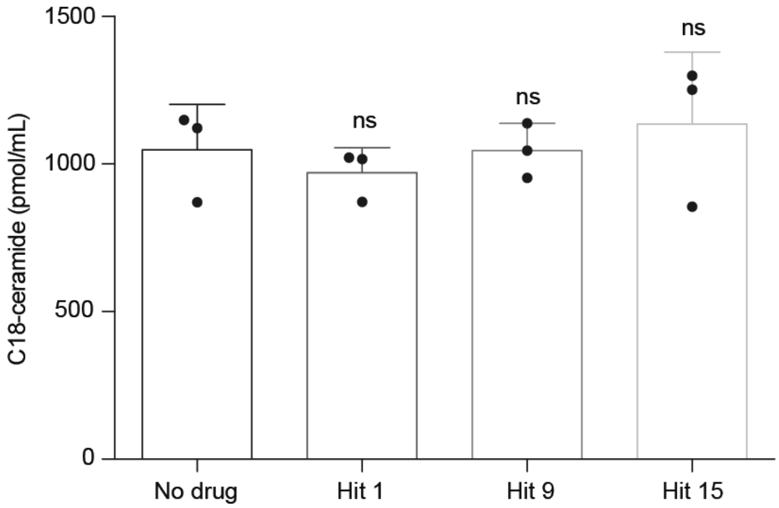

Supplementary Figure 9. Time dependence evaluation of ergosterol 3β-D-glucoside (erg-glc) accumulation inside *C. neoformans* wilt-type H99 cells exposed to Hits 1 (white bars), 9 (light grey), and 15 (dark grey) at 100 μM until 48 hours. The total amount of erg-glc detected intracellularly (LC-MS) was similar from 3 to 24 hours incubation with each compound, however a significant decrease was observed after 48 hours treatment. Statistical analysis was performed by Two-way ANOVA followed by Tukey's multiple comparison test for each compound. Results were expressed as mean ± SD of n=3 independent experiments.

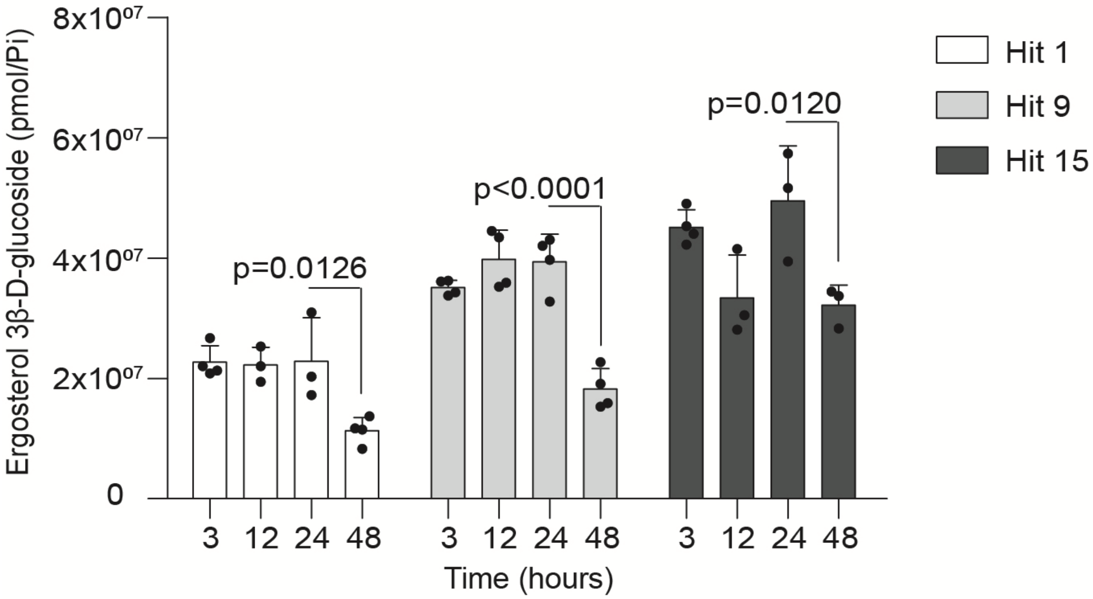

Supplementary Figure 10. 2Fo-Fc electron density of Hit 1 (**a**) and (**b**) Hit 9.

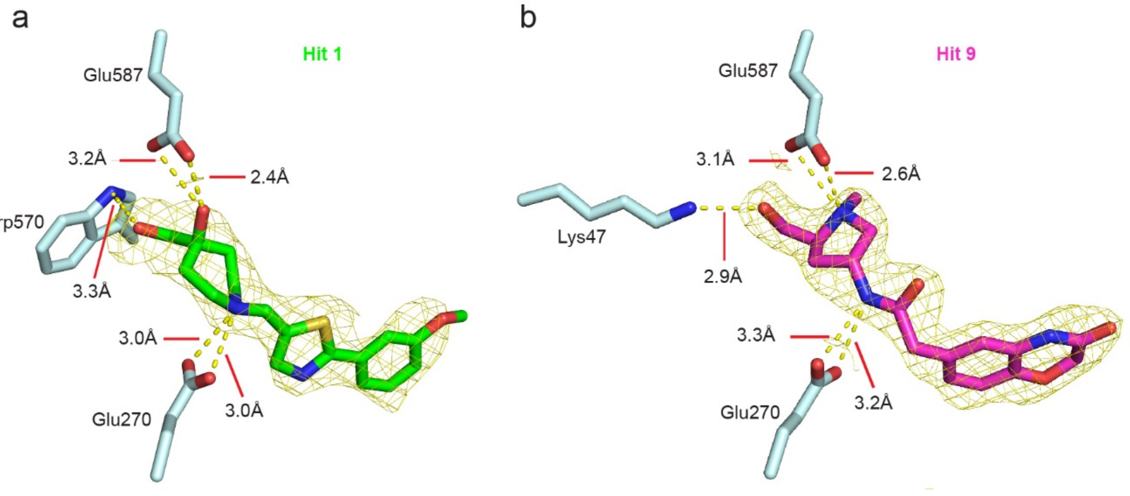

References

1. Courtney, S. M.; Hay, P. A.; Scopes, D. I. Pharmaceutically active benzoxazole, benzthiazole, and benzimidazole acid derivatives. Patent WO2004046122 (2004).
2. Delhomel, J. F.; Perspicace, E.; Majd, Z.; Parroche, P.; Walczak, R. *N*-{[2-(Piperidin-1-yl)phenyl](phenyl)methyl}-2-(3-oxo-3,4-dihydro-2H-1,4-benzoxazin-7-yl)acetamide derivatives and related compounds as ROR-gamma modulators for treating autoimmune diseases.Patent WO2018138362 (2018).
3. Miyashita, A.; Iijima, C.; Higashino, T. Studies on pyrazolo[3,4-d]pyrimidine derivatives. XVIII. Facile preparation of 1*H*-pyrazolo[3,4-d]pyrimidin-4(5*H*)-ones. *Heterocycles*. **31**, 1309-1314 (1990).
