# Supplementary material for: Structure and inhibition of Cryptococcus neoformans sterylglucosidase to develop antifungal agents
Source: Nat Commun. 2021 Oct 7;12:5885. doi: 10.1038/s41467-021-26163-5 (PMC8497620; doi:10.1038/s41467-021-26163-5)
Supplement: Supplementary file 4 — Legend for Supplementary Data 1 [file 41467_2021_26163_MOESM4_ESM.docx]

Supplementary Data 1

Pdb files of erg-glc, C6-NBD-glcCer and fungal glcCer docked with *C.neoformans* Sgl1
