# Supplementary material for: Structure and inhibition of Cryptococcus neoformans sterylglucosidase to develop antifungal agents
Source: Nat Commun. 2021 Oct 7;12:5885. doi: 10.1038/s41467-021-26163-5 (PMC8497620; doi:10.1038/s41467-021-26163-5)
Supplement: Supplementary file 5 — Reporting Summary [file 41467_2021_26163_MOESM5_ESM.pdf]

nature portfolio

Corresponding author(s): Michael Airola and Maurizio Del Poeta

Last updated by author(s): Sep 3, 2021

Reporting Summary

Nature Portfolio wishes to improve the reproducibility of the work that we publish. This form provides structure for consistency and transparency in reporting. For further information on Nature Portfolio policies, see our Editorial Policies and the Editorial Policy Checklist.

Statistics

For all statistical analyses, confirm that the following items are present in the figure legend, table legend, main text, or Methods section.

n/a	Confirmed	
☐	☒	The exact sample size (*n*) for each experimental group/condition, given as a discrete number and unit of measurement
☐	☒	A statement on whether measurements were taken from distinct samples or whether the same sample was measured repeatedly
☐	☒	The statistical test(s) used AND whether they are one- or two-sided<br>*Only common tests should be described solely by name; describe more complex techniques in the Methods section.*
☒	☐	A description of all covariates tested
☒	☐	A description of any assumptions or corrections, such as tests of normality and adjustment for multiple comparisons
☐	☒	A full description of the statistical parameters including central tendency (e.g. means) or other basic estimates (e.g. regression coefficient) AND variation (e.g. standard deviation) or associated estimates of uncertainty (e.g. confidence intervals)
☐	☒	For null hypothesis testing, the test statistic (e.g. *F*, *t*, *r*) with confidence intervals, effect sizes, degrees of freedom and *P* value noted<br>*Give P values as exact values whenever suitable.*
☒	☐	For Bayesian analysis, information on the choice of priors and Markov chain Monte Carlo settings
☒	☐	For hierarchical and complex designs, identification of the appropriate level for tests and full reporting of outcomes
☒	☐	Estimates of effect sizes (e.g. Cohen's *d*, Pearson's *r*), indicating how they were calculated

Our web collection on statistics for biologists contains articles on many of the points above.

Software and code

Policy information about availability of computer code

Data collection	Dock v6.9
Data analysis	Coot 0.8.9.1<br>Phenix 1.15rc1-3423<br>CCP4 7.0.000<br>UCSF Chimera 1.14<br>GraphPad Prism 5.01

For manuscripts utilizing custom algorithms or software that are central to the research but not yet described in published literature, software must be made available to editors and reviewers. We strongly encourage code deposition in a community repository (e.g. GitHub). See the Nature Portfolio guidelines for submitting code & software for further information.

Data

Policy information about availability of data

All manuscripts must include a data availability statement. This statement should provide the following information, where applicable:

- Accession codes, unique identifiers, or web links for publicly available datasets
- A description of any restrictions on data availability
- For clinical datasets or third party data, please ensure that the statement adheres to our policy

Coordinates and structure factors have been deposited in the Protein Data Bank under accession codes 7LPO [https://doi.org/10.2210/pdb7LPO/pdb], 7LPP [https://doi.org/10.2210/pdb7LPP/pdb], 7LPQ [https://doi.org/10.2210/pdb7LPQ/pdb]. The mass spectrometry proteomics data have been deposited to the Proteo-meXchange Consortium via the PRIDE partner repository with the dataset identifi-er PXD02498. Pdb files of erg-glc, C6-NBD-glcCer and fungal glcCer docked

with Cn Sgl1 are included with this manuscript. EGCase II and cerezyme structures displayed in Figures 2d and 2e can be found under the accession codes 2OSX [https://10.2210/pdb2OSX/pdb] and 6TJQ [https://10.2210/pdb6TJQ/pdb], respectively. Source data are provided as a source data file.

Field-specific reporting

Please select the one below that is the best fit for your research. If you are not sure, read the appropriate sections before making your selection.

☒ Life sciences ☐ Behavioural & social sciences ☐ Ecological, evolutionary & environmental sciences

For a reference copy of the document with all sections, see nature.com/documents/nr-reporting-summary-flat.pdf

Life sciences study design

All studies must disclose on these points even when the disclosure is negative.

Sample size	Sample sizes are given in the manuscript. All assays were repeated at least three times, which is sufficient to derive error bars, p values, and statistical significance.
Data exclusions	No data was excluded from the analyses.
Replication	All experimental findings were confirmed with at least three independent replicates as detailed in Methods or Figure Legends. All attempts at replication were successful.
Randomization	All enzymatic assays were conducted with wild type Cn Sgl1 in order to test the effect of deletion of active site residues. No randomization is needed for these assays. For structure determination, 5% of the reflections were randomly included in the Rfree calculations, as per standard X-ray crystallography refinement procedures.
Blinding	No blinding was performed as the bias from prior knowledge of samples in case of biochemical and imagining experiments described in the study is considered limited. All samples including controls were analyzed in exactly the same manner.

Reporting for specific materials, systems and methods

We require information from authors about some types of materials, experimental systems and methods used in many studies. Here, indicate whether each material, system or method listed is relevant to your study. If you are not sure if a list item applies to your research, read the appropriate section before selecting a response.

Materials & experimental systems

n/a	Involved in the study	
☒	☐	Antibodies
☐	☒	Eukaryotic cell lines
☒	☐	Palaeontology and archaeology
☐	☒	Animals and other organisms
☒	☐	Human research participants
☒	☐	Clinical data
☒	☐	Dual use research of concern

Methods

n/a	Involved in the study	
☒	☐	ChIP-seq
☒	☐	Flow cytometry
☒	☐	MRI-based neuroimaging

Eukaryotic cell lines

Policy information about cell lines

Cell line source(s)	Human lung adenocarcinoma cell line A549 (American Type Culture Collection #CCL-185). Cryptococcus neoformans H99 cells (American Type Culture Collection #208821) and Δsgl1 mutant strain Cryptococcus neformans (Rella et al., 2015)
Authentication	A549 cell line was purchased from ATCC (CCL-185). C. neoformans var. grubii serotype A H99 cell line was purchased from ATCC (208821). Cryptococcus neformans Sgl1 mutant strain was generated by Rella et al. (2015) (locus number CNAG_05607 in C. neoformans var. grubii serotype A genome database).
Mycoplasma contamination	Early A549 cell passage number 7-9 was used to ensure prevent genetic drift and mycoplasma contamination developed after too many passages.
Commonly misidentified lines (See ICLAC register)	*Name any commonly misidentified cell lines used in the study and provide a rationale for their use.*

Animals and other organisms

Policy information about studies involving animals; ARRIVE guidelines recommended for reporting animal research

Laboratory animals	CBA/J female mice aged 3 to 4 weeks old.
Wild animals	This study did not involve wild animals.
Field-collected samples	This study did not involve samples collected from the field.
Ethics oversight	Mouse experiments were performed in full compliance with a protocol approved by Stony Brook University (IACUC number 341588) and in compliance with the United States Animal Welfare Act (Public Law 98-198). The experiments were carried out in facilities accredited by the Association for Assessment and Accreditation of Laboratory Animal Care.

Note that full information on the approval of the study protocol must also be provided in the manuscript.
